# Reminiscences

**Published:** 1886-02

**Authors:** C. G. Von Bonhorst


### Reminiscences.

BY C. G. VON BONHORST.

That various operations on the teeth were performed in Ages shrouded in the dim mists of antiquity, we have no reason to doubt. In the *jaws* of many disentombed skeletons of ancient date, evidences of filling as well as loss of teeth, by extraction, have been found, also artificial dentures remaining in the jaws, proving, that in some form or other, dentistry was known and practiced by the Ancients; nor is it surprising that such should be the case, great attention was paid to the person in those days. It may be that the instruments used for dental operations, were not as perfectly adapted to their intended uses as those of the present day, but no doubt they answered the purpose about as well as some of those made fifty years ago. I can well remember my first introduction to dental implements, how very crude they were, compared with what we now have. Forceps that broke as many teeth as they pulled out. To say nothing of the old fashioned cant hook, as many called it. I remember too, an old dentist, who, when he wanted to insert upper front teeth, did not go to a dental depot, and select such teeth as might be required for the case, but went to his butcher and asked him when he was going to kill another sheep, as he wanted some more teeth. These he cut the roots from, then drilled holes in them, and inserted them on hickory pivots. But he was a genius; graduated from a barber shop to the dental chair. There are many other strange fancies that I could refer to: About 1844, a so-called dentist came to Pittsburg, and astonished the natives by hawking from house to house ready made dentures. What would the profession think of such a proceeding nowadays? The invention consisted of teeth, mounted on flat, gold wire, about an eighth of an inch wide, and about half as thick, having from one tooth up to six. It was only applicable to the loss of the six upper front teeth—and then only when the roots remained in the jaw. The fixture was kept in place by gold pivots inserted in the roots—no roots, no teeth. It did not quite fill the bill.

The most of the filling done then was with tin foil, as it was so much cheaper than gold. Amalgam, too, pure, and unadulterated, which got as black as your hat, was greatly in vogue, but still, teeth were saved, although in mourning. The first experience with gold foil I ever had, was with some made by old Mr. B. F. Uffington, an old English gentleman, then manufacturing gold foil and gold leaf, in Pittsburg. John B. Dunlevy learned his trade under him. It was what now would be termed soft foil, and made good fillings. About this time, the then great problem of air chambers in dental plates came to my notice. And I saw a great variety of ways and shapes, which all promised to make a plate stick so tight it would pull the patient's ear back; but I also discovered the fact, that whenever I got an impression that was correct, it made little difference whether the plate had an air chamber or not.

About this time, a dentist, whose name shall not be mentioned, astonished the toothless by claiming to have a system of inserting *teeth without plates.* However, his plan was different from the one now advertised; his teeth were made much like S. S. White's biscuited teeth for porcelain work are now made, with roots, and the roots ribbed crosswise of the tooth. His method was to select a tooth the size as near as possible, of the one to be extracted, then introduce it in the jaw, when the other was extracted, bind it to the natural teeth, and allow the gums to heal —I am sorry to say, I never heard of any remaining permanently. I had some experience with a *gutta percha* covered *tin plate,* warranted not to cut in the eye. On the recommendation of a good old dentist in Cincinnati, since passed away, I put up an upper and lower set of teeth on this new base, for the wife of a prominent judge in Virginia. Some months after, he wrote me that the plates stuck first rate, but thought they were getting black. Shades of Moses; so I thought when I again saw them. I, however, with the best grace I could assume, substituted a set of continuous gum teeth. Calling to remembrance, the time when the lamented John T. Toland owned the Cincinnati Dental Depot, and published the *Dental Register,* the all absorbing question of "*Dental Pups,*" as John used to head line them, came prominently before the profession: and what is more, is staying with it. As to the different methods of treating them, that is the pulps, each man had his own particular method, whilst some had none at all. The question is still agitating the public mind. I remember well what a furore the announcement of the achievement of contour fillings created, it bid fair to swallow all other discoveries in dentistry. Building up with gold, an old tooth, with top, bottom and sides gone. Yet by the simple "rule of three," little retaining points, the mass of gold restoring size and shape of original tooth, was to be held in situ for all time. This operation does not seem to be so popular as it was, especially with the patient.

One great improvement I must advert to, viz.: The Rubber Dam, an invention which does more for the dentist who conscientiously feels inclined to do good filling, than all other inventions ever made for that purpose. No dentist can ignore the fact, although I do not think it is required in all cases. Yet I would not do without it. It begets confidence in the patient, as well as the operator, besides making it easier to operate, without fear of wetting the filling. All credit to Dr. Barnum, although I don't believe he has made as much out of his invaluable discovery as his namesake, P. T., did out of the unfortunate Jumbo. We hear of many new inventions in dentistry—every little while something new—all for the comfort of patient and operator. This speaks well for the profession. But there are men who have never availed themselves of the privileges afforded them in the use and possession of many of the improvements of the present day, but go along in the same old rut they have traveled in for years, oblivious of the fact that much younger members of the profession are in the front ranks, bearing aloft the motto, "Excelsior," whilst many others are denying them selves the benefits of attending those great and good social reunions, where dentist can speak to dentist as to a brother, where there is no strife save that which emulates some brother dentist in the good work.

To my old time associates in the profession, let me say, that although thousands of miles separate us, memory often overlaps the space, and I am again with those among whom I spent many of my most happy days.

